# Numerical Training Videos and Early Numerical Achievement: A Study on 3-Year-Old Preschoolers

**DOI:** 10.3390/brainsci12010088

**Published:** 2022-01-11

**Authors:** Alessandro Cuder, Marta Vidoz, Chiara De Vita, Sandra Pellizzoni, Maria Chiara Passolunghi

**Affiliations:** Department of Life Sciences, University of Trieste, 34128 Trieste, Italy; alessandro.cuder@phd.units.it (A.C.); martavidoz@gmail.com (M.V.); chiaradv@hotmail.it (C.D.V.); spellizzoni@units.it (S.P.)

**Keywords:** learning, mathematics, preschoolers, training, videos, counting, cardinality

## Abstract

Early numerical abilities predict later math achievement and could be improved in children by using various training methods. As the literature on the use of training videos to develop numerical abilities is still surprisingly scant, the aim of the present study was to test the efficacy of a numerical training video on the development of counting and number line knowledge in 3-year-old preschoolers. Far transfer effects to cardinality and working memory were also examined. The study involved 86 children randomly assigned to two intervention groups: a numerical training group exposed to videos on counting and number lines; and a control group exposed to videos on colors and animal names in a foreign language. After the video training, there was an improvement in the numerical training group’s counting skills, but not in their number line knowledge, and this improvement persisted six months later. The numerical training group also showed a far-transfer enhancement of cardinality six months after the intervention. Based on our results, numerical training videos could be effective in helping to enhance early numeracy skills in very young preschoolers.

## 1. Introduction

In an increasingly technological and numeracy-based society, children’s mathematical schooling is fundamental to their long-term general development. The literature suggests that individuals’ numerical abilities predict their future educational, occupational, and financial success, positively influencing outcomes such as employment [1,2], salary size [3], and socioeconomic status [4,5]. Although numeracy is a strong predictor of success in life, several studies have demonstrated that around 20% of students show low numerical skills, and from 4% to 14% of children and adolescents exhibit a learning deficit in at least one mathematical area [6,7,8,9].

Given the impact of mathematical competence on individuals and societies, it seems crucial to assess numerical abilities from a very early age in order to take action to prevent potential learning difficulties in the sphere of mathematics, from pre-school onwards. Several studies have suggested that mathematical learning is sustained by a set of general and specific cognitive skills, defined as ‘precursors’ [10,11,12,13].

### 1.1. Domain-Specific Precursors

Domain-specific numerical skills specifically predict future mathematical performance [14,15,16]. They include counting, or the ability to establish a one-to-one relationship between objects in a set and their numerical representations [17,18]. Learning to count has been found to play a fundamental part in sustaining later mathematical learning in both primary [19,20,21] and secondary school [22]. Typically, this skill begins to develop from the age of two and, although children are able to recite the words in order, they do not understand that the aim of counting is the quantification [23,24,25,26]. Another domain-specific skill, related to counting, is the ability to understand cardinality, i.e., that the last word counted stands for the total number of items in the counted set [23,27,28,29]. Implicit in this ability there is the understanding that number words correspond to unique and specific quantities [30], which is a strong predictor of later mathematical achievement [27,31,32,33]. Literature shows that cardinality skill develops from the third to the fourth year of age following the full acquisition of the counting process [24,25,34]. Another domain-specific precursor is number line knowledge, which refers to the ability to form a mental representation of a graduated line on which each number corresponds to a position [35,36]. This skill seems to be related to a wide range of numerical abilities [37] and, as suggested by a recent meta-analysis [38], it is recognized as a strong predictor of mathematical learning.

### 1.2. Domain-General Precursors

While domain-specific abilities are specifically associated with mathematical learning, domain-general skills relate to abilities that predict children’s academic success in other school subjects as well [39,40]. One domain-general skill is working memory (WM), which seems to influence academic success in a number of disciplines. WM is a cognitive system comprising several subsystems that handle both verbal and visuospatial information [41,42,43,44]. Additionally, there is a distinction between high-control WM processes that require concurrent storage, processing, and effortful mental activity, and low-control WM processes involved in the retainment of small amounts of information that are subsequently recalled [45,46]. Literature shows that WM is associated with children’s performance in calculus, problem solving and mathematics [47,48,49]. Visuo-spatial WM is needed to acquire a basic knowledge of numbers [46,50,51], while verbal WM seems to be more useful later on, for the acquisition of more complex mathematical abilities [46,47,51], such as math fluency and word problem solving [20,52].

### 1.3. Numeracy Training Studies

Having established the predictive role of domain-specific and domain-general precursors, research has focused on developing interventions to boost the skills associated with mathematical achievement. Few studies on training measures to enhance numerical abilities have been conducted on 3-year-olds to date (e.g., [53]), but the literature suggests that exposing preschool children to suitably-designed materials can enhance abilities linked to mathematical learning. To give some examples, domain-specific training has reportedly been successful in promoting numerical skills such as counting [53,54,55,56,57,58], cardinality [28,29], and number line knowledge [53,55,59]. As regards the training effectiveness for age groups, interventions on counting and cardinality seem to have provided positive outcomes already at three years of age [28,53], while number line trainings have been proved particularly useful in the last years of preschool [55,58,59]. Mixed results were obtained, on the other hand, with interventions addressing domain-general WM skills [54,56,60,61]. According to recent meta-analyses [61,62], WM training interventions seem to produce short-term effects that fail to generalize to arithmetical skills. Most of preschool WM interventions available in the literature were conducted on samples who had an average age ranging from four [63] to five years [54,56,64]. It is still unclear whether WM abilities can be enhanced at an early age, however, and this possibility is worth exploring.

Domain-specific or domain-general training studies have traditionally been based on face-to-face interventions (especially for younger children), with operators engaging in educational activities with a group of children, or else on a computer-based modality for single individuals. These types of training promote the active participation of the children involved, in both computer-based activities (e.g., [57,59]) and small group activities (e.g., [53,58,65]). Past interventions targeting preschoolers usually proposed content embedded in playful activities requiring some degree of numerical processing (e.g., [54,56,65]). For instance, board games usage is commonly reported in literature for developing counting and number line abilities because it exposes children to the order of numbers and numerical quantities [66]. Other studies developed entertaining videogames in which numerical skills were trained in the context of playful challenges bringing improvements in counting, cardinality and number line knowledge [57,59]. These interventions commonly lasted from two to five weeks with meetings scheduled twice a week.

Nowadays, children can be passively exposed via child-friendly apps on tablets or mobile phones to a variety of videos developed to entertain or promote learning. According to a survey conducted on parents in the US [67], children from 2 to 4 years of age were exposed to screens for about two and half hours a day. Nearly three in four respondents (73%) reported that their children’s screen time was spent mainly watching television and videos, with a large amount of the time (37%) occupied by videos on online platforms (see also [68]). Despite such a pervasive trend, little is known about the real effects of exposing children to educational videos in terms of their content acquisition. Part of the literature suggests that video learning could be difficult for certain age groups [69]. In particular, some studies have shown that very young children (from the first to the second year of age) can exhibit deficit in learning through videos and transferring the assimilated knowledge in different testing contexts [70,71,72,73]. However, the effects of exposure to videos with numerical content on the development of preschoolers’ numerical skills remain unclear.

The limited literature on the topic dates back to the early 1970s, when the focus was on assessing the educational impact of TV programs (e.g., [74,75]). An example is the TV program “Sesame Street”, popular in the US, which targeted preschool children in an effort to boost several early life skills. Numerous studies found exposure to this show associated with positive effects on linguistic, mathematical, and everyday living skills [70,71,72,76]. These investigations commonly lasted for several months and preschoolers were shown educational content embedded in several episodes of Sesame Street at home or at school. Progress was then assessed with different tools and compared to the viewing time reported by parents and teachers. A meta-analysis on several such studies and reports from various countries also confirmed that watching Sesame Street was associated with positive outcomes on linguistic and mathematical abilities in children with different cultural backgrounds [77]. Alongside research generally examining the educational effectiveness of TV programs, there are studies that investigated the role of crucial variables in the learning of numerical skills from watching videos. For instance, Lauricella and colleagues [78] found that exposure to numerical videos had positive effects on children’s seriation abilities providing the characters were perceived as meaningful. Another study by Aladé and colleagues [79] found that a video-based training could enhance measurement skills and promote far-transfer effects on dissimilar tasks. Schroeder and Kirkorian [80] also demonstrated that children perform better on quantity discrimination when they are passively exposed to numerical videos than when interfacing with interactive applications. In short, although preschoolers tend to watch videos every day, at home and elsewhere [67,68], the literature on passive learning through video exposure is still surprisingly scant.

### 1.4. The Present Study

In the light of the above considerations, the aim of the present study was to investigate the effects of exposure to videos with numerical content on the development of early numerical abilities in a sample of 3-year-old preschoolers. Most of the previously-mentioned studies used face-to-face training and interactive programs to improve early numerical abilities, and none examined the effects of exposure to numerical videos on domain-specific abilities (such as counting, cardinality, and number line knowledge) and domain-general abilities (such as WM) at the same time. Given how online video consumption is increasing among today’s preschoolers [67,68], this trend could be exploited to convey evidence-based interventions employing commonly-used devices. For the present study, we therefore prepared numerical training videos, and assessed their impact on the development of early numerical skills (i.e., counting, cardinality and number line knowledge) and low-control WM abilities. First, we hypothesized that counting ability could be fostered through passive exposure to videos showing children the enumeration process, as reported elsewhere (e.g., [56,58]). Second, we hypothesized that exposure to numerical videos could enhance number line knowledge, in line with reports that a correct linear representation of numbers could be developed by exposing children to the spatial arrangement of numbers and quantities on a straight line (e.g., [65]). We also expected counting video training to have an effect on children’s understanding of cardinality, as evidence suggests that counting is a precursor to cardinality [81,82]. Finally, we explored the effects of numerical video training on the development of low-control WM skills, an issue on which the literature is inconsistent [54,56,60,61]: we did not expect our numerical training videos to improve the children’s WM.

## 2. Materials and Methods

### 2.1. Participants

The initial sample selected for this study included 91 preschoolers, 3 years of age, attending nursery schools in Northeast Italy. With the schools’ approval, parents were contacted to obtain their consent to their children’s participation. The parents of 5 children refused, so the final sample in the pretest phase consisted of 86 children (40 females; M_age_ = 43.3 months, SD_age_ = 3.21). Participants were randomly assigned to one of two groups: a numerical training group (*n* = 43; M_age_ = 42.9 months, SD_age_ = 2.95); and an active control group (*n* = 43; M_age_ = 43.7 months, SD_age_ = 3.43). Missing data and the outliers were handled with listwise deletion rather than run imputation methods to avoid biased score estimations. In the pretest and post-test evaluation one participant (*n* = 1) was removed as an outlier because of his emigrational background and his difficulties to comprehend the verbal WM task. At the follow-up, 12 participants were removed from the analysis (*n* = 3 due to outlier scores in numeracy abilities evaluation and *n* = 9 due to dropout from the study). All other participants attended all the training sessions. All participants were Caucasian and their socio-economic status (judging from the school records) was middle class. A written informed consent form was signed by each child’s parents before they took part in the study. Children were asked for their verbal consent to their participation in the activities prior to the assessment and training sessions. The study was conducted in accordance with the Declaration of Helsinki, the ethical guidelines of the Italian Association of Psychology, and the ethical code of the Italian Register of Professional Psychologists.

### 2.2. Procedure

The study was conducted in four phases.

#### 2.2.1. Pretest Phase

In the pretest phase, the children were tested on their domain-specific and domain-general abilities in two sessions (20 min each). Vocabulary knowledge and attentional tasks were also administered as control measures to check for any differences between the two groups’ cognitive levels. Counting ability, understanding of cardinality, and number line knowledge were used to assess their early numerical (domain-specific) skills, and WM was assessed as a domain-general ability.

#### 2.2.2. Training Phase

In the subsequent training phase, participants were exposed to the numerical training or active control videos in small groups (3 to 5 children). The training sessions were held twice a week. Twelve sessions were scheduled, during each of which children were shown two videos on the same topic. The active control group was shown videos about colors and animal names.

#### 2.2.3. Posttest Phase

In a third, posttest phase, the children were tested on their early numerical abilities and WM.

#### 2.2.4. Follow-Up Phase

Five months later, at the beginning of the second year of nursery school we added a follow-up assessment and tested children on counting ability, cardinality understanding, and number line knowledge only.

### 2.3. Pretest Control Measures

#### 2.3.1. Receptive Vocabulary

WPPSI-III [82] was used to assess the children’s word knowledge. They were shown 38 sheets of paper with 4 printed images and asked to indicate which of the four images corresponded to the word the researcher was saying. The task was interrupted after 5 consecutive errors. The total score was the sum of the correctly recognized images and could range from 0 to 38.

#### 2.3.2. Selective Attention

A task was adapted from an attention subtest in the BVN 12-18 battery [83]. The test involved watching a video in which several visual stimuli were randomly presented on a screen at a rate of one picture per second. The children were asked to clap their hands whenever a target stimulus (e.g., the sun) appeared on the screen. The total score, calculated as the sum of correctly identified target stimuli, could range from 0 to 20.

#### 2.3.3. Visuospatial Low-Control WM

The task used to assess visuospatial WM involved the child remembering a toy frog’s movements within a 3 × 3 matrix, touching the cells previously indicated by the researcher [84]. The test included four levels of increasing difficulty, with two sets of movements for each level. It was interrupted when a child made two mistakes on the same level of difficulty. Scores were calculated as the sum of the positions correctly recalled. The total score could range from 0 to 8.

#### 2.3.4. Verbal Low-Control WM

This task involved presenting lists of commonly-used words and asking the child to repeat each list immediately after hearing it, recalling the words in the same order of presentation [84]. The test included four levels of difficulty for a total of eight tests. The task was interrupted when a child made two mistakes on the same level of difficulty. Scores were calculated as the sum of the words correctly recalled in the right order. The total score could range from 0 to 8.

### 2.4. Pretest and Posttest Numeracy Measures

#### 2.4.1. Counting

Counting skills were assessed with a task adapted from an enumeration subtest in the Numerical Intelligence Battery [85]. The children were asked to count forwards from 1 to 20 and their total score corresponded to the highest number counted correctly.

#### 2.4.2. Cardinality

A task adapted from Le Corre and Carey [25] was used to assess the children’s understanding of cardinality. They were randomly presented with nine cards showing from 1 to 9 different objects. For each card, they were asked first to count the objects, then to say how many objects there were on the card. They scored one point if they correctly counted the objects and answered the examiner’s question correctly without needing to start the counting process again. Scores could range from 0 to 9.

#### 2.4.3. Number Line Knowledge

A task adapted from Laski and Siegler [86] was used. The children were shown a line with five equally-spaced notches and the numbers “1” and “5” at the ends. They were then shown random numbers from 1 to 5 and asked to place them along the number line. They scored one point for each number correctly placed on the line. Total scores in this task could range from 0 to 5.

### 2.5. Characteristics of the Intervention

#### 2.5.1. Numerical Training Group Intervention

We first established the features the videos needed to have to suit the purposes of our intervention. We wrote video scripts with two puppet characters (a bear and a tiger) playing games that involved counting and number lines, with numbers ranging from 1 to 10. For instance, the videos that focused on counting involved enumerating objects or simply pronouncing numerical sequences, while the number line videos presented number symbols or sets of objects arranged along a graduated number line. The scenes were recorded with a reflex camera (Nikon D3100, with a Nikon 18–55 mm f3.5 lens), then the raw videos were dubbed and edited. This procedure was used to create six videos, three on counting and three on number lines (see Table 1).

#### 2.5.2. Control Group Intervention

Videos for the control group were selected on the YouTube platform, choosing among those dubbed in a foreign language (English) and suitable for 3-year-old preschoolers (e.g., videos about color names, see Table 1). These videos were edited to match the duration and resolution of our numerical training videos, obtaining six videos, three about colors and three about animals. We used foreign language videos because we decided to offer contents, unrelated to numerical activities, which could be perceived by children as entertaining and engaging, with the same educational value of numerical training videos.

## 3. Results

Table 2 shows the descriptive and univariate test results (from MANOVA) for the two groups.

### 3.1. Pretest Assessment

To check for any differences between the two groups at the baseline, we ran a multivariate analysis of variance (MANOVA) with group (numerical training group vs control group) as a fixed factor and pretest measures (receptive vocabulary, selective attention, visuospatial WM, verbal WM, counting, cardinality and number line knowledge) as dependent variables. A measure of effect size was used to compare the differences between the groups’ pretest scores. Cohen’s criteria [87] were used to classify the effect size: small effect (η_p_^2^ = 0.01), medium effect (η_p_^2^ = 0.06), large effect (η_p_^2^ = 0.14). Effect sizes (Cohen’s *d*) for pairwise comparisons are also reported; small effect *d* = 0.20; medium effect *d* = 0.50; large effect *d* = 0.80.

The results of the MANOVA showed no significant effect of group (Wilks’ Lambda = 0.941, *F*(7,77) = 0.69, *p* = 0.678, η_p_^2^ = 0.974), meaning that the two groups did not differ statistically in the pretest phase. Univariate tests showed no statistically significant difference in their receptive vocabulary *F*(1,83) = 0.79, *p* = 0.376, η_p_^2^ = 0.009, selective attention *F*(1,83) = 0.01, *p* = 0.958, η_p_^2^ = 0.000, visuospatial WM *F*(1,83) = 0.12, *p* = 0.732, η_p_^2^ = 0.001, verbal WM *F*(1,83) = 0.80, *p* = 0.374, η_p_^2^ = 0.010, counting *F*(1,83) = 1.15, *p* = 0.286, η_p_^2^ = 0.014, cardinality *F*(1,83) = 1.97, *p* = 0.164, η_p_^2^ = 0.023 or number line knowledge *F*(1,83) = 1.42, *p* = 0.237, η_p_^2^ = 0.017. In short, there were no statistically significant differences between the two groups at pretest.

### 3.2. Posttest Assessment

We calculated the gains (posttest minus pretest) in the children’s early numerical abilities (counting, cardinality, number line knowledge) and WM scores. This analytical strategy—comparing gains from pre- and post-training assessments—had been used in several previous studies (e.g., [56,88,89,90]). We ran a MANOVA using group as the fixed factor and gain in scores as dependent variables. We also applied Bonferroni’s adjusted post-hoc pairwise comparisons of the gains. To compare the gains made between the two groups, η_p_^2^ and effect sizes (Cohen’s *d*/Hedges’ *g*) were used for post-hoc pairwise comparisons.

The results of the MANOVA showed a significant main effect of group (Wilks’ Lambda = 0.725, *F*(5,80) = 6.07, *p* < 0.001, η_p_^2^ = 0.275), suggesting statistically significant differences between the two groups at posttest. The univariate analysis showed a statistically significant difference between the groups for counting *F*(1,84) = 25.8, *p* < 0.001, η_p_^2^ = 0.235, pointing to different effects of the two interventions. Bonferroni’s adjusted pairwise post-hoc comparisons indicated a significant effect of the numerical training on counting ability compared with the control group (M_diff_ = 5.00, *p* < 0.001, *d* = 1.095). No statistically significant difference emerged between the groups for visuospatial WM *F*(1,84) = 2.03, *p* = 0.158, η_p_^2^ = 0.024, verbal WM *F*(1,84) = 0.73, *p* = 0.39, η_p_^2^ = 0.009, cardinality *F*(1,84) = 0.19, *p* = 0.661, η_p_^2^ = 0.002, or number line knowledge *F*(1,84) = 1.16, *p* = 0.285, η_p_^2^ = 0.014.

### 3.3. Follow-Up Assessment

We calculated the gains (follow-up minus pretest) from pretest to follow-up in the children’s counting, cardinality, and number line knowledge scores, conducting a MANOVA with group as a fixed factor and gains in scores as dependent variables.

The results showed a significant main effect of group (Wilks’ Lambda = 0.737, *F*(3,70) = 8.31, *p* < 0.001, η_p_^2^ = 0.263) suggesting statistically significant differences between the two groups. The univariate analysis showed statistically significant differences for counting *F*(1,72) = 14.9, *p* < 0.001, η_p_^2^ = 0.171 and cardinality *F*(1,72) = 11.5, *p* = 0.001, η_p_^2^ = 0.138, pointing to different effects of the two interventions. Compared with the control group, Bonferroni’s adjusted pairwise post-hoc comparisons indicated long-term enhancement effects of the numerical training on the children’s counting ability (M_diff_ = 4.237, *p* < 0.001, *d* = 0.9197), and understanding of cardinality (M_diff_ = 2.661, *p* = 0.001, *d* = 0.791). No statistically difference emerged between the numerical training and control groups on number line knowledge *F*(1,72) = 0.05, *p* = 0.814, η_p_^2^ = 0.001.

To sum up, we found a statistically significant improvement in the counting task at posttest in the numerical training group by comparison with the control group. Other differences in the two groups’ gains on cardinality, number line knowledge and WM were not statistically significant. We also found differences at the follow-up comparison between the two groups, which were significant for counting and cardinality, but not for number line knowledge.

## 4. Discussion

The aim of the present study was to assess the effectiveness of a passive video-based training for promoting specific numerical abilities in 3-year-old children. After 12 sessions of exposure, our analysis of posttest performance gains showed enhanced counting skills in the numerical training group compared with the control group. This outcome replicates previous study findings on the positive effect on counting skills of various types of stimulation, such as board games (e.g., [65]) and computer-based activities (e.g., [57]). The significant gains seen in the numerical training group’s counting abilities also persisted five months later, demonstrating long-lasting effects of the intervention. This could be because the training videos were designed to offer considerable benefits in a development age when children are predisposed to acquiring counting skills [17,91,92]. Passive viewing of numerical training videos could therefore be a good way to enhance counting abilities in 3-year-olds.

On number line knowledge, our numerical training group did not perform significantly better than the control group, neither at posttest nor at follow-up. This outcome contrasts with other reports of improvements gained by exposing children to number line training interventions [53,55,56,59]. One reason for our findings could lie in our sample’s age, as the children may have been too young to benefit from the training. In fact, a recent study [93] found that preschool children performed poorly in even the simplest number line estimates and, unsurprisingly, number line training interventions described in the literature were conducted on children more than 3 years old [53,55,59]. Passive video stimulation might also be inadequate for developing number line knowledge. Future intervention studies could test other methods that have proved successful in the literature, such as board games (e.g., [55]) and computer-based activities (e.g., [59]).

Potential far transfer effects on cardinality and WM were also examined. As concerns the children’s understanding of cardinality, the numerical training group’s gains did not differ significantly from the control group’s at posttest. This result is consistent with previous studies involving training programs that used counting to enhance children’s understanding of cardinality [28,29]. On the other hand, our numerical training group showed an improvement in this area, compared with the control group, at the follow-up five months later. This was probably a far transfer effect of their counting training, which could have helped them to develop stronger cardinality-related skills. The literature has consistently shown that number list knowledge and counting skills are fundamental to understanding the quantities associated with number words [23,30,81].

No far transfer effects were seen on visuospatial or verbal low-control WM. This is in line with previous research (e.g., [56,60]) focusing on WM interventions, which generated mixed results [54,56,60,61], and any short-term effects failed to generalize to numerical abilities [61,62]. Our results extend the evidence on the inability of numerical training videos to influence WM in young preschoolers.

### Limitations and Future Directions

More research is needed to overcome the limitations of the present study. For a start, we were unable to distinguish between the effects of counting and number line training videos because we exposed the children in the numerical training group to both types of material. Future studies should replicate our results while separately examining the effects of training these and other early numerical abilities. We also used only one training modality. Moreover, we should recognize that cardinality and number line tasks could have been too challenging for some children, especially at the pretest assessment. This could have biased our results lowering discriminant power of instruments or affecting children motivation in executing proposed tasks. Future research should include and compare different methods, such as face-to-face and computer-based training interventions, to explore any differences in their effectiveness.

## 5. Conclusions

To conclude, we found a positive effect of exposure to numerical training videos on counting, but not on number line knowledge. Our analyses also showed a significant benefit on the children’s understanding of cardinality five months after they had received the numerical training, whereas it had no influence on their WM. Our findings confirm the importance of considering videos as a useful way to strengthen early numerical skills in preschool age. Unlike training interventions discussed in the literature, our study focused on an age group not usually considered for intervention studies on children’s numerical abilities. We also conducted a follow-up assessment of the children’s numerical abilities six months after the end of the numerical training, which showed lasting effects of the intervention on their counting skills and understanding of cardinality. Given the role of numerical skills in predicting individual and collective educational, economic and financial success, our findings may have relevant practical implications. For instance, videos could be a simple and effective way to develop and disseminate training interventions on a large scale, given the popularity and accessibility of video streaming platforms among preschoolers [67,68,94]. They could be used to deliver entertaining, targeted and effective interventions in deprived settings where numerical skills are undertrained, and thereby prevent the risk of children developing difficulties and negative attitudes to mathematics as they grow older [95,96].

## Figures and Tables

**Table 1:** Topic, duration in minutes and content description of the training videos.

**Training Videos**			
	**Topic**	**Duration in Minutes**	**Content Description**
1	Counting	4	Characters were involved in counting activities from 1 to 9. Example: puppets had to count mushrooms’ white dots.
2	Counting	4	Characters were involved in counting activities from 1 to 10. Example: puppets had to count fruit sets that were placed inside a case.
3	Counting	4	Characters were involved in counting activities from 1 to 10. Example: puppets counted candies placed inside a plastic bottle.
4	Number line	4	Characters were involved in arranging numbers on a number line from 1 to 5. Example: puppets had to sort some jars with numbers printed on them in a number line.
5	Number line	4	Characters were involved in arranging numbers on a number line from 1 to 5. Example: puppets had to sort digits on a number line.
6	Number line	4	Characters were involved in arranging numbers on a number line from 1 to 10. Example: puppets had to sort ten stones with digits printed on them on a number line.
**Active Control Group Videos**			
	**Topic**	**Duration in Minutes**	**Content Description**
1	Colors	4	Characters were involved in naming colors. Example: puppets had to name the colors of flower’s petals (red, blue, green, yellow).
2	Colors	4	Characters were involved in naming colors. Example: puppets had to name the colors of fruits (red, orange, green, purple, yellow).
3	Colors	4	Characters were involved in naming colors. Example: puppets had to name the colors of candies (red, orange, purple, yellow).
4	Animals	4	Characters were involved in naming animals. Example: puppets had to name jungle animals (tiger, monkey, elephant, parrot, giraffe).
5	Animals	4	Characters were involved in naming animals. Example: puppets had to name wood animals (wolf, owl, deer, fox).
6	Animals	4	Characters were involved in naming animals. Example: puppets had to name sea animals (whale, shrimp, turtle, shark).

**Table 2:** Reliabilities (R), mean scores (M), standard deviations (SD), minimum (Min.) and maximum (Max.) scores, and univariate test results (*F* and *F*_gains_ from MANOVA) in the different measures for the two groups at the three time points (pretest, posttest, follow-up). ** *p* < 0.01, *** *p* < 0.001.

**Pretest Assessment**								
**Measures**	**R**	**Training Group** **(*n* = 42)**		**Control Group** **(*n* = 43)**		**Min.**	**Max.**	***F***
		**M**	**SD**	**M**	**SD**			
Receptive vocabulary	0.94	10.7	3.24	11.3	3.07	1	17	0.79
Selective attention	0.92	16.2	4.30	16.2	2.85	5	20	0.01
Visuospatial WM	0.80	2.44	1.18	2.51	1.03	0	4	0.12
Verbal WM	0.89	3.02	0.87	2.88	0.54	0	4	0.80
Counting	0.83	7.84	5.19	8.81	4.47	0	20	1.15
Cardinality	0.84	2.30	2.88	3.02	2.64	0	9	1.97
Number line knowledge	0.75	1.40	1.14	1.67	1.32	0	5	1.42
**Post-test Assessment**								
**Measures**	**R**	**Training Group** **(*n* = 42)**		**Control Group** **(*n* = 43)**		**Min.**	**Max.**	***F*_gains_**
		**M**	**SD**	**M**	**SD**			
Visuospatial WM	0.80	2.67	0.94	3.09	0.97	0	5	2.03
Verbal WM	0.89	3.38	0.76	3.12	0.50	0	5	0.73
Counting	0.83	12.5	4.41	8.51	4.59	0	20	25.8 ***
Cardinality	0.84	4.58	3.51	5.02	2.76	0	9	0.19
Number line knowledge	0.75	1.65	1.15	1.58	1.18	0	5	1.16
**Follow-Up Assessment**								
**Measures**	**R**	**Training Group** **(*n* = 33)**		**Control Group** **(*n* = 41)**		**Min.**	**Max.**	***F*_gains_**
		**M**	**SD**	**M**	**SD**			
Counting	0.83	14.2	4.86	10.8	5.81	0	20	14.9 ***
Cardinality	0.84	6.85	2.76	5.07	2.99	0	9	11.5 **
Number line knowledge	0.75	1.24	0.94	1.71	1.19	0	5	0.05

## Data Availability

The data presented in this study are available on request from the corresponding author. The data are not publicly available due to privacy.
